# Songbird population trajectories diverge under simulations of conifer encroachment versus removal in a sagebrush ecosystem

**DOI:** 10.1002/eap.70228

**Published:** 2026-04-06

**Authors:** Elise C. Zarri, Jason D. Tack, Joseph T. Smith, Scott L. Morford, Thomas E. Martin, David E. Naugle

**Affiliations:** ^1^ Numerical Terradynamic Simulation Group, University of Montana Missoula Montana USA; ^2^ U.S. Fish and Wildlife Service, Habitat and Population Evaluation Team Missoula Montana USA; ^3^ Montana Cooperative Wildlife Research Unit University of Montana Missoula Montana USA; ^4^ W.A. Franke College of Forestry and Conservation University of Montana Missoula Montana USA

**Keywords:** Brewer's Sparrow, forecast, habitat management, productivity, restoration, territory occupancy

## Abstract

Woody plant encroachment into grasslands and shrublands is a global phenomenon that negatively impacts ecosystem services and wildlife populations. North American sagebrush ecosystems have experienced widespread degradation from encroaching conifers, leading to losses of sagebrush‐obligate wildlife. Removal of encroaching trees is a primary restoration method, but whether management actions can influence wildlife populations at management‐relevant scales is rarely investigated. We studied a local Brewer's Sparrow in the Medicine Lodge Valley of southwest Montana to understand how their territory occupancy and nest success were impacted by tree and shrub cover. From 2019 to 2022, our data collection resulted in 1161 mapped territories and 449 nests, which we used to build models of territory occupancy and reproductive productivity relative to tree and shrub cover. We then used tree and shrub cover extracted from historical imagery to estimate population size and productivity 70 years in the past. Finally, using models of tree growth and expansion, we estimated population size and reproductive productivity under two simulated scenarios 30 years in the future: with and without restoration through conifer removal. We observed that tree cover has more than tripled at the study site since 1954, which our models predicted has caused an almost 25% decrease in the local population size and a 35% decline in offspring production. In a future scenario where tree removal is conducted in areas with <20% tree canopy cover after 30 years, we predict population size and offspring production will likely remain stable. Alternatively, if tree cover is allowed to increase unabated, our simulations predict a potential population decline of 60%, with similar losses to offspring production. We observed a stark divergence in the potential futures of the local Brewer's Sparrow population and implications for the species at large. Continuing tree encroachment drastically decreases the available habitat and causes a steep decline in population size. However, proactive and continuing management of encroaching trees can alleviate further losses in a species that has already experienced significant range‐wide declines. We also highlight the importance of encroaching trees as a previously underappreciated conservation risk for sagebrush avifauna.

## INTRODUCTION

Humans have altered and degraded natural landscapes across the globe, with negative consequences for biodiversity and wildlife populations (Ceballos et al., 2015, 2017; Díaz et al., 2019). Grasslands and shrublands are at significant risk, with woody plant encroachment being a major driver of habitat loss (Nackley et al., 2017; Sala & Maestre, 2014; Stevens et al., 2017). Trees and shrubs are expanding into rangeland habitats, primarily due to anthropogenic shifts in disturbance regimes, such as wildfire suppression and high grazing pressure (Archer et al., 2017). Woody plant encroachment has wide‐ranging effects on ecosystem functions and services, including the loss of livestock forage, alteration of hydrologic processes and carbon storage, and reductions in native grassland and shrubland flora and fauna (Anadón et al., 2014; Eldridge et al., 2011; Sala & Maestre, 2014; Soliveres et al., 2014). The removal of encroaching woody plants is a common restoration technique; however, its effectiveness in reversing habitat degradation and restoring ecosystem services is not universal. Variations in the type of habitat encroached (e.g., grassland, savanna, or shrubland), removal technique, and stage of encroachment impact outcomes (Ding & Eldridge, 2023a, 2023b). Thus, follow‐up studies are critical to ensure that restoration through woody plant removal produces the intended effects.

Sagebrush shrublands in western North America have declined drastically since European settlement and continue to face widespread threats from invasive annual grasses and encroaching native conifers (Doherty et al., 2024; Noss et al., 1995; Schroeder et al., 2004). Tree encroachment into North American rangelands has been ongoing since the 1800s (Archer et al., 2017; Burkhardt & Tisdale, 1976; Miller et al., 2008; Miller & Rose, 1999; Tausch et al., 1981), but the scope of encroachment has only recently been fully measured with the increasing use of remotely sensed data (Knick et al., 2003; Morford et al., 2024). We now understand that almost 450,000 ha of intact sagebrush have been encroached by trees just since 2000 (Mozelewski et al., 2024), and millions of hectares have been lost since European settlement (Miller et al., 2008; Tausch et al., 1981). As trees encroach, some rangeland ecosystem functions and services are lost, including forage production for cattle and wildlife, soil water availability, and wildlife habitat (Maestas et al., 2021; Morford et al., 2022; Roundy, Young, et al., 2014). Conifer removal is a common strategy to restore rangelands, prevent further loss of intact sagebrush ecosystems, and attempt to reverse species decline (Doherty et al., 2022; Reinhardt et al., 2020). As we begin to understand the vast extent of sagebrush habitat that has been lost to encroaching trees, the following questions arise: What role does tree encroachment play in the historical and ongoing losses of sagebrush wildlife? Can restoration through conifer removal change these trajectories?

Sagebrush‐obligate wildlife have faced concurrent declines as shrublands have been lost and degraded (Knick et al., 2003; Leipold et al., 2024; Rowland et al., 2006). The Greater Sage‐Grouse (*Centrocercus urophasianus*), whose population has declined by 78% range‐wide since 1960 (Prochazka et al., 2024), exemplifies the losses of sagebrush wildlife and has inspired significant conservation action (Connelly et al., 2011). Other species, such as pygmy rabbits (*Sylvilagus idahoensis*), pronghorn (*Antilocapra americana*), and sagebrush‐obligate songbirds, have also faced population declines as sagebrush habitats have degraded (Leipold et al., 2024, Rowland et al., 2006). Many sagebrush‐associated species are sensitive to tree cover, and research has shown that conifer removal can increase abundance and productivity (Donovan et al., 2024; Larrucea & Brussard, 2008; Olsen et al., 2021; Zarri et al., 2024). The exact reasons for tree avoidance are unknown, but potential explanations include increased predation from forest‐associated species (LaManna et al., 2015; Tewksbury et al., 1998) and trees that serve as perch sites for avian predators (Young et al., 2023). However, the demonstrable population‐level benefits of conifer removal remain largely unquantified for species other than Greater Sage‐Grouse (Olsen et al., 2021).

We often assume that conifer removal will restore functional sagebrush ecosystems that provide habitat for sagebrush‐associated wildlife, but the consequences of alternative management decisions are rarely quantified at management‐relevant scales. Here, we used a sagebrush‐obligate songbird experiencing range‐wide declines, the Brewer's Sparrow (*Spizella breweri*), to understand the historical impact of conifer encroachment and to predict future outcomes under alternative management scenarios. Brewer's Sparrows are emblematic of sagebrush‐obligate wildlife. Like many species in the biome, they avoid tree cover and select territory with high shrub cover (Donnelly et al., 2017; Kumar et al., 2024; Tack et al., 2023; Zarri et al., 2024). Recent evidence shows that they are more abundant and have better reproductive outcomes when encroaching trees are removed (Zarri et al., 2024), but how this translates to population‐level outcomes has not been measured. Brewer's Sparrows are an ideal indicator species for the health of the sagebrush biome because they are highly sensitive to annual changes in vegetation due to their short lifespans and low site fidelity (Rotenberry et al., 2020; Wiens et al., 1986). Similar to many songbirds, they are visible and easy to monitor for both population size and reproductive output (Ralph et al., 1993). Our objectives were to (1) quantify how 70 years of tree encroachment into an otherwise intact sagebrush landscape has impacted a local population of Brewer's Sparrows; and (2) predict future population outcomes under simulated alternative management scenarios. To accomplish these objectives, we built empirical models linking Brewer's Sparrow territory occupancy and reproductive rates to habitat structure based on data from a four‐year field study and remotely sensed fractional vegetation cover. Using these models, we predicted population size and reproductive productivity under a set of detailed simulations of past and future habitat structure based on historical imagery and simple models of tree encroachment and infill.

## MATERIALS AND METHODS

### Study site

**FIGURE 1:**
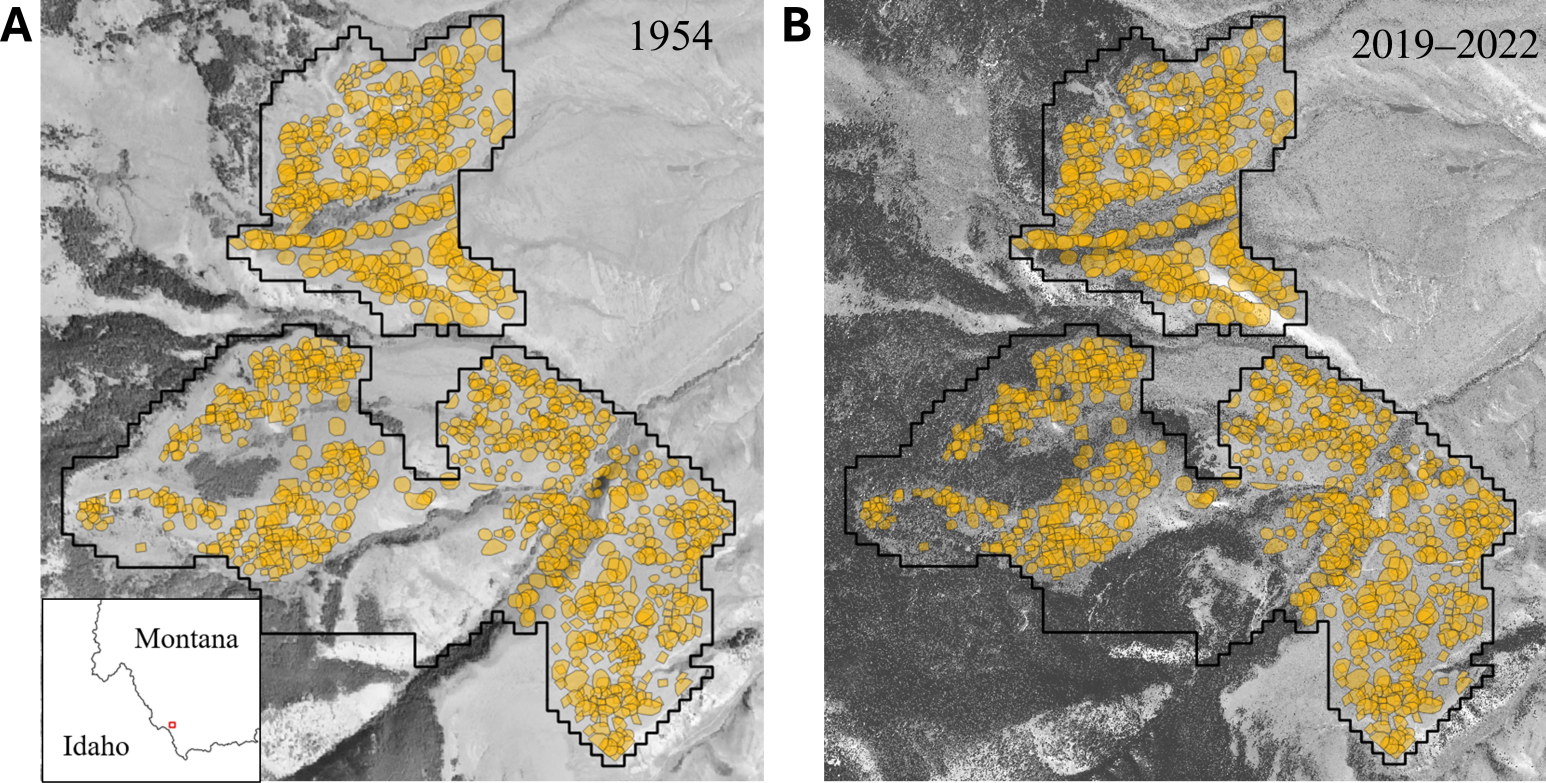
Mapped Brewer's Sparrow territories (yellow polygons) from 2019 to 2022 are plotted against aerial imagery in the current time period (A) and historically available data (1954, B). Inset map shows the location of the study site in southwest Montana.

We conducted this study in the Medicine Lodge Valley (44.84126, −113.04998) of southwest Montana, USA, during the breeding seasons of May–August of 2019–2022. The vegetation at the study site was primarily mountain big sagebrush (*Artemisia tridentata vaseyana*), encroached by Douglas fir (*Pseudotsuga menziesii*) and junipers (*Juniperus* spp.) from the adjacent dense forest. Conifers have been encroaching on this site since at least 1954, based on historical imagery (Figure 1), with no significant impact from invasive annual grasses or direct human development. Areas at early stages of encroachment were selected for tree removal based on percent tree cover and evidence of historical treelessness. Almost 4 km^2^ of conifer removal was conducted in 2017–2018 by hand‐felling to prevent damage to shrub cover. All trees extending above the shrub layer were removed. Matched areas with similar preremoval tree cover, elevation, slope, aspect, sagebrush height, and vigor were left as control plots (details in Zarri et al., 2024). We excluded riparian areas from the analysis because they constitute a distinct ecological system, are not used by Brewer's Sparrows, and exhibit a different relationship between tree and shrub cover than the surrounding shrublands. A crew of eight field technicians collected data each summer across a total area of 8.62 km^2^, split into 12 plots for ease of surveying.

### Territories and nests

**FIGURE 2:**
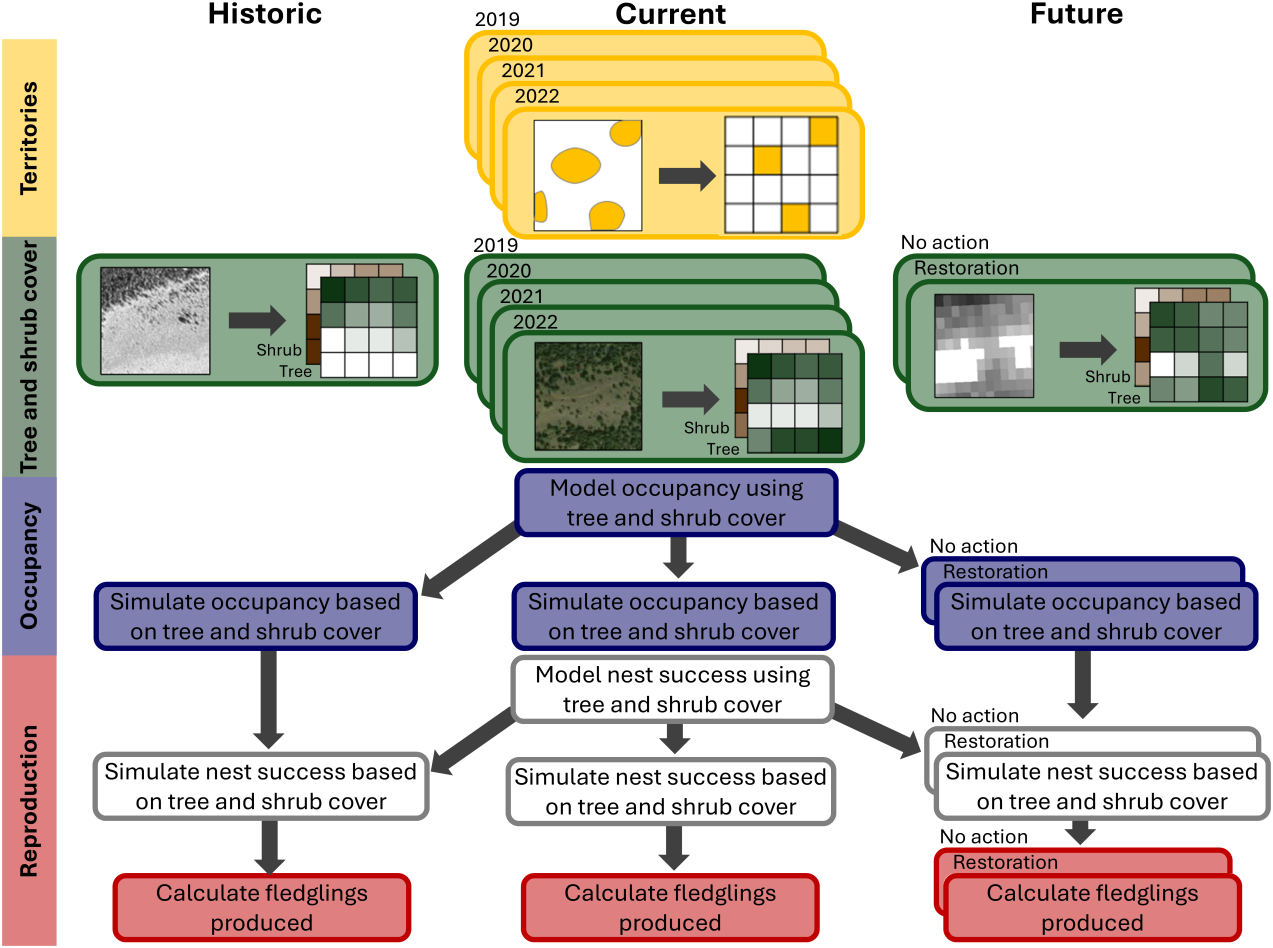
Flow chart depicting how data were analyzed, starting with mapped Brewer's Sparrow territories in the current time period (2019–2022), which were converted to an occupancy grid with cell sizes equal to the mean size of a Brewer's Sparrow territory. Then, tree and shrub cover was collected from remotely sensed datasets in the current period, and tree and shrub cover was estimated in the historic (1954) and future (2050) scenarios. We created a model of territory occupancy based on tree and shrub cover and ran 1000 simulations for each scenario to estimate the effect of tree and shrub cover change on population size. We then created a model of nest success and used the same simulation framework, assuming that each territory had a nest. Finally, we used fledgling production data to calculate the number of fledglings produced in each simulation for each scenario.

We collected Brewer's Sparrow territory data using full plot surveys three to five times throughout the breeding season, along with daily observations made while searching for nests. For each observation, we noted whether the bird was seen or heard and visually estimated its distance and direction from the observer to mark its location on a map. We used handheld GPS units with numbered points every 50 m to pinpoint bird locations. Countersinging allowed us to identify the number of territories and their boundaries (Ralph et al., 1993; Zarri et al., 2024). At the end of each breeding season, we used plot surveys, daily bird observations, and nest locations to draw maps of all the territories across the study site (Figure 1). Next, we converted the territory maps into a grid of occupied and unoccupied cells so that territory occupancy could be simulated under other scenarios. Using average territory area as the cell size, we overlaid a grid across the study site (Figure 2). For each territory, we calculated the centroid and assigned it to the corresponding grid cell. In rare cases where >1 territory centroid occupied the same cell, we reassigned territories to adjacent unoccupied cells containing the largest proportion of the territory area.

We found and monitored Brewer's Sparrow nests to collect detailed reproductive data (details in Zarri et al., 2024). A crew of eight field technicians spent 4–6 h per day searching for nests using parental behavior and systematic searching of sagebrush plants (Martin & Geupel, 1993). Once nests were found, we checked them every 1–3 days to record their contents and fate.

All field surveys and bird handling were conducted under Montana Fish Wildlife and Parks permits 2019‐010‐W, 2020‐008‐W, 2021‐021‐W, and 2022‐033‐W, U.S. Fish and Wildlife Service permit MB791101‐0, U.S. Geological Survey federal bird banding permit 21635, and University of Montana Institutional Animal Care and Use Committee Animal Use Proposals 061‐18 and 046‐20.

### Tree and shrub cover

#### Current landscape

We estimated tree and shrub cover at the study site in 2019–2022 for use as covariates in the models of occupancy and nest success. For each grid cell, we extracted annual estimates of the fractional cover of trees and shrubs during 2019–2022 from the Rangeland Analysis Platform (RAP; Allred et al., 2021; Figure 2), a remotely sensed map of fractional vegetation cover for the coterminous United States.

#### Historical landscape

To estimate historical vegetation conditions relevant to Brewer's Sparrow occupancy and reproductive success, we first derived estimates of historical tree cover. We downloaded aerial images and tree cover data of the study site from 1954 using the Landscape Explorer tool (Morford et al., 2024). From these data, we extracted percent tree cover at 30‐m resolution, masked out riparian areas, and summarized the mean tree cover values for each grid cell. Because methods have not been developed to estimate shrub cover from historical aerial imagery, we inferred historical shrub cover by leveraging the relationship between tree and shrub cover observed in the contemporary RAP data. Tree cover is a significant, nonlinear driver of change in shrub cover (Appendix S1: Figure S1; Miller et al., 2000), so we left shrub cover unchanged if tree cover in a given grid cell was within 5% of its contemporary RAP value. For all other grid cells, we used a k‐nearest neighbors (KNN) approach to generate values for historical shrub cover. We used the Euclidean distance in elevation, slope, heat load index (McCune & Keon, 2002), and tree cover to identify the three most similar grid cells in the contemporary landscape and imputed the mean shrub cover value for the historical landscape.

#### Future landscapes

To project tree and shrub cover 30 years into the future, we simulated two contrasting scenarios: *no action* and *restoration*. In the *no action* scenario, we assumed that no future conifer management would occur while trees continued to encroach. We started with a 1‐m binary map of tree presence/absence from 2017 based on NAIP imagery (Morford et al., 2024), and identified all “source tree” pixels. To simulate tree encroachment and infill, we created 200 m buffers around the original 1 m tree pixels to represent seed dispersal zones (Fogarty et al., 2022; Vander Wall, 2023). To align with coarser resolution data from RAP, we aggregated the 1 m buffer data to 30 m, creating a tree expansion layer. Using RAP data from 1990 to 2020, we calculated an average tree encroachment rate of 0.67% cover per year, adding up to 20% over 30 years. We applied this rate to all areas within the 30 m pixels overlapping the tree expansion layer. Pixels outside the buffer areas were assumed to retain their 2017 tree cover values due to a lack of seed source. We imputed shrub cover using the same 5% rule and KNN imputation approach as used for the historical shrub cover.

The *restoration* scenario assumes ongoing conifer removal efforts. For this scenario, we used post‐treatment tree cover data from 2018 as a starting point and simulated future tree cover growth using the same encroachment method described above. However, we assumed that all areas with <20% tree cover would undergo hand‐felling of encroaching conifers after 30 years, removing all tree cover while leaving shrub cover intact, and again imputed the shrub cover as described above.

### Occupancy model

Next, we modeled Brewer's Sparrow territory occupancy as a function of remotely sensed tree and shrub cover. We extracted shrub and tree cover within each cell for use as covariates. We also expected that vegetation in a larger neighborhood around the grid cell would impact territory occupancy (Chalfoun & Martin, 2007; Johnson, 1980), so we derived the mean tree and shrub cover using square buffers at 70, 140, 280, 500, and 1000 m around each grid cell. We masked the riparian areas in both the grid cells and buffers. Using the grid of occupied and unoccupied cells built from territory maps in 2019–2022 as the response variable, we fit a set of generalized linear mixed models with a binomial error distribution, all of which included random effects of year and plot. We built our models in a stepwise fashion by first testing univariate models of tree and shrub cover with a combined model and selecting the top model using corrected Akaike information criterion (AIC_c_). We then tested a set with each buffer size and finally tested the top buffer size model with those including topographic variables such as elevation, slope, and heat load. We selected the final, most parsimonious model using AIC_c_ (Burnham & Anderson, 2002).

We then used the top occupancy model to predict occupancy in the historic, current, and future scenarios. We ran 1000 simulations for each scenario, predicting occupancy in each grid cell using a binomial draw with the probability of occupancy derived from the top occupancy model. From the simulations, we were able to calculate the likelihood of occupation in each grid cell across all simulations and the number of Brewer's Sparrow pairs per square kilometer by averaging over the study site in each simulation. We modeled territory occupancy in the current time period to compare simulated results with observed data.

### Reproductive models

Next, we assessed how tree and shrub cover impacted reproductive rates, including clutch size, nest success, and proportion of eggs fledged. These rates represent the potential mechanisms through which food availability and predation risk impact songbird populations. For example, clutch size or proportion of eggs fledged may increase with shrub cover if insect prey is associated with dense sagebrush (Haab et al., 2023; Rotenberry & Wiens, 1989). If predation is important, we expect nest success to decrease as tree cover and forest‐associated predators increase (Young et al., 2023). We quantified tree and shrub cover within 13 radii (30–100 m at 10‐m intervals, and 200, 300, 400, 500, and 1000 m) around each nest using RAP data, excluding riparian areas. We again used a successive stepwise model‐building procedure for each reproductive component. We used generalized linear mixed models with Poisson error distributions for clutch size models, and binomial error distributions for proportion of eggs fledged and nest success models. All models included a random effect of year. We first tested model sets with fixed effects of shrub cover, both linear and quadratic, at various buffer sizes. We selected a top model using AIC_c_ and then tested a set of models that added tree cover at various buffer sizes. Finally, we added topographic variables and used AIC_c_ to select the top model for each reproductive component.

For each iteration of the simulations in each scenario (historic, current, future *restoration*, and future *no action*), we assumed that every occupied grid cell produced a single nesting attempt. We used the centroid of the cell as a nest location and extracted tree and shrub cover in circular buffers around the nest at the scale indicated by the top model. We then predicted nest outcomes at each occupied cell based on the top nest success model and calculated the number of fledglings produced for each successful nest using the predictions from the successive vital rate models. Because neither clutch size nor the proportion of eggs that fledged were influenced by tree or shrub cover, we drew these values randomly from the observed distribution. Each iteration of the simulation yielded a predicted number of fledglings produced from a single nest attempt per territory, which we converted to fledglings per kilometer by averaging over all cells in the study site.

Data analysis was conducted using QGIS (QGIS Development Team, 2024), Google Earth Engine (Gorelick et al., 2017), and R (R Core Team, 2025). Generalized linear mixed models were fit using the lme4 package (Bates et al., 2015) in R.

## RESULTS

### Territories and nests

We found a total of 1161 Brewer's Sparrow territories and 449 nests from 2019 to 2022. The mean territory size was 4869 ± 101 m^2^ (SE), so we superimposed a 70 × 70 m grid over the study site.

### Tree and shrub cover

**FIGURE 3:**
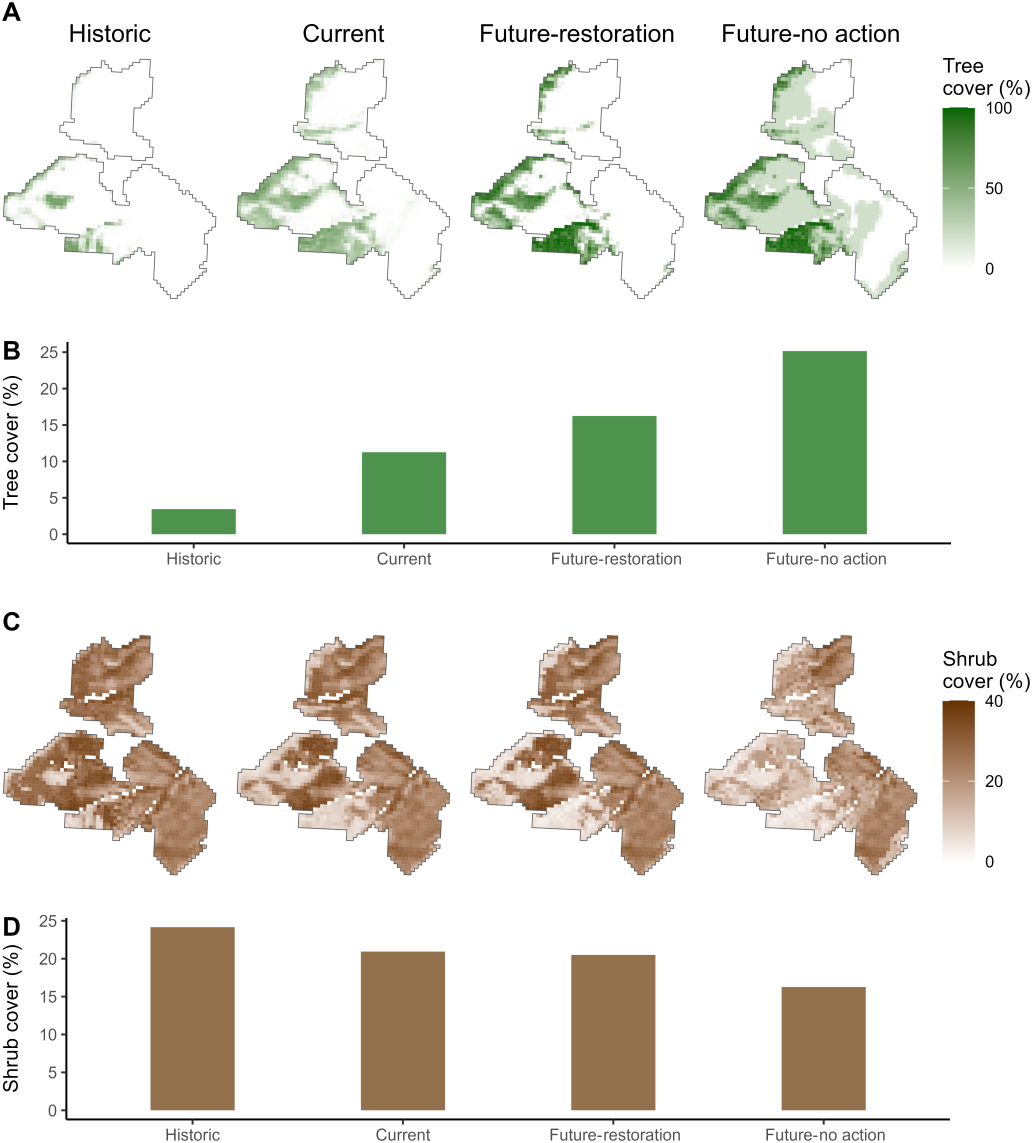
Maps of the study site showing fractional tree (A) and shrub cover (C), and calculated mean tree (B) and shrub cover across the four scenarios analyzed, including historic (1954), current (2020), and two future scenarios (2050) based on simulated restoration via management of conifer cover <20% or no action over the next 30 years.

We observed that the mean tree cover across the study site increased from 3.5% in 1954 to 11.3% in 2019 (Figures 1 and 3A). If no restoration action is taken in the next 30 years, our simulation predicts that tree cover will increase to 25.2% (Figure 3A,B). However, if restoration action is taken, the simulated tree cover increases slightly to 16.2% as currently forested areas experience an infill.

Shrub cover decreased from 24.2% in 1954 to 20.9% in 2019 and was simulated to decrease to 16.3% in the future scenario with no action taken (Figure 3C,D). However, restoration through tree removal resulted in the stabilization of shrub cover, with 20.5% cover predicted in 30 years.

### Occupancy model

The top territory occupancy model included tree and shrub cover within the 70‐m grid cell, tree cover in a 70‐m buffer around the cell, and elevation (Table 1). Occupancy increased with shrub cover (β ± SE = 0.062 ± 0.008, *p* = <0.001) and elevation (0.459 ± 0.081, *p* = <0.001) and decreased with tree cover in the grid cell (−0.020 ± 0.012, *p* = 0.103) and in a 70‐m buffer (−0.061 ± 0.012, *p* = <0.001).

**TABLE 1:** Corrected Akaike information criterion (AIC_c_) model selection results for the final model set from the successive model‐building procedure from Brewer's Sparrow territory occupancy models.

Model	*K*	AIC_c_	ΔAIC_c_
Shrub + tree + tree 70 + elevation	7	5122.34	0
Shrub + tree + tree 70 + elevation + heat	8	5122.55	0.21
Shrub + tree + tree 70 + elevation + heat + slope	9	5123.88	1.54
Shrub + tree + tree 70	6	5154.90	32.56
Shrub + tree + tree 500	6	5165.21	42.87
Shrub + tree + tree 1000	6	5165.24	42.90
Shrub + tree + tree 140	6	5165.35	43.01
Shrub + tree	5	5166.94	44.60
Shrub + tree + tree 280	6	5168.86	46.52
Tree	4	5222.75	100.41
Shrub	4	5234.72	112.38

*Note*: “Tree” and “Shrub” are the percent cover of that vegetation type within the 70‐m^2^ grid cells. Numbers after the tree or shrub indicate cover in a buffer around the grid cell. All models included random effects of year and plot.

**FIGURE 4:**
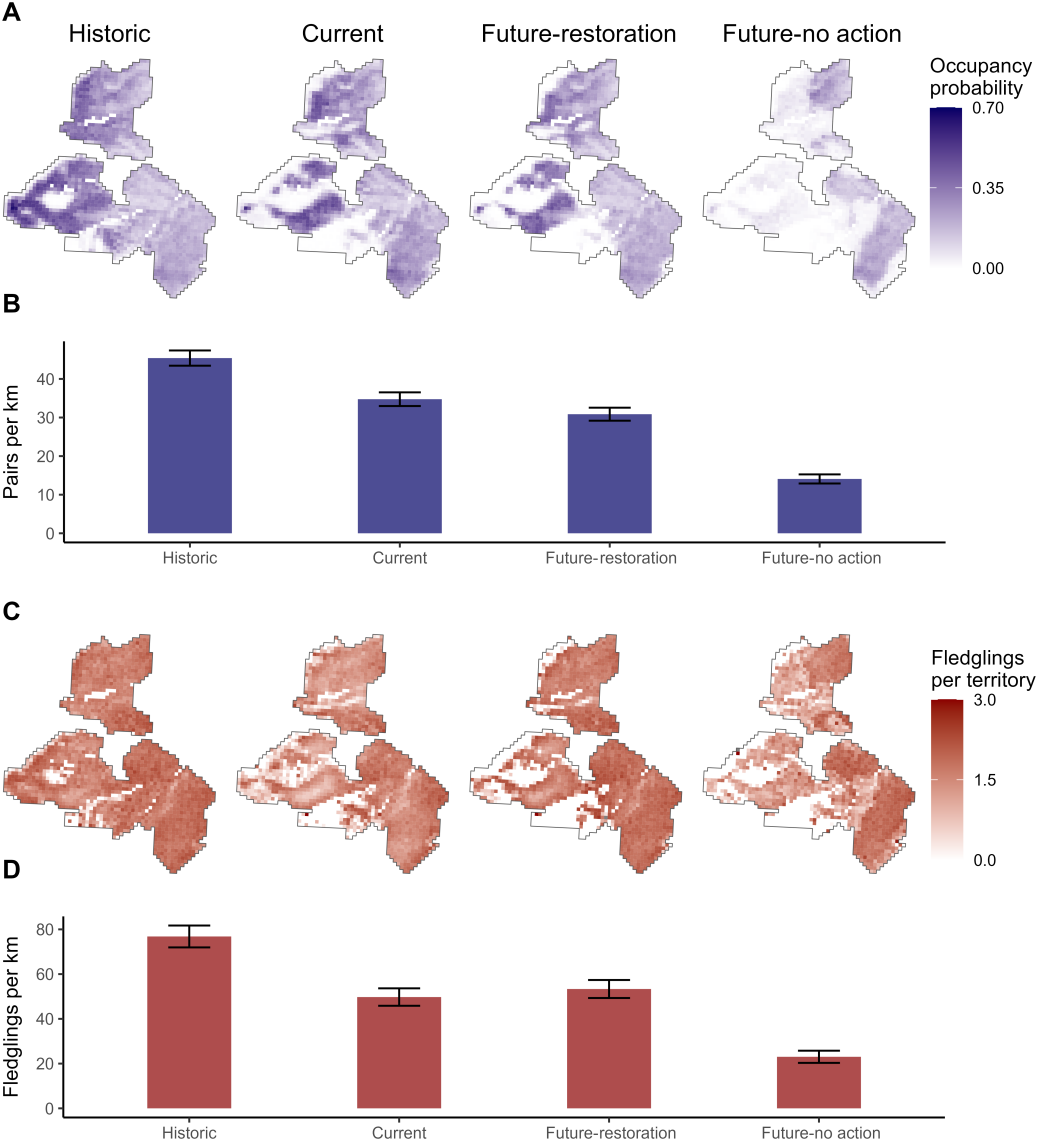
Maps of the study site depicting Brewer's Sparrow territory occupancy probability (A) and fledglings per territory (C). Mean ± SD of the estimated pairs per square kilometer (B) and fledglings per square kilometer (D) were derived by summing across the study site for each of 1000 simulations among the four scenarios analyzed, including historic (1954), current (2020), and two future scenarios (2050) based on simulated restoration via management of conifer cover <20% or no action over the next 30 years.

Our occupancy simulations predicted 34.80 ± 1.76 (SD) pairs km^−1^ in the current period, equal to 300 pairs total across the study site. This aligns closely with our observed 36.04 ± 3.76 pairs km^−1^ in 2019–2022 from the territory maps. In 1954, our simulations suggested that the population was approximately 30% higher at 45.42 ± 1.87 pairs km^−1^ or 392 pairs total. In the future *no action* scenario, our simulations predict that the Brewer's Sparrow population will decrease by nearly 60% to 14.10 ± 1.18 pairs km^−1^ or 121 pairs total. However, under the *restoration* scenario, the simulation predicts a population decrease of only 11% to 30.81 ± 1.63 pairs km^−1^ or 266 pairs total (Figure 4A,B).

### Reproductive models

Neither clutch size nor proportion of eggs fledged were associated with tree or shrub cover (Appendix S1: Tables S1 and S2), but nest success was affected by tree cover within 30 m, a quadratic relationship with shrub cover within 300 m, and slope (Table 2). The probability of nest success decreased with tree cover (β ± SE = −0.074 ± 0.035, *p* = 0.033) and slope (−0.042 ± 0.022, *p* = 0.055) and peaked at moderate values of shrub cover (shrub 300 = 0.076 ± 0.269, *p* = 0.778; shrub 300^2^ = −0.004 ± 0.006, *p* = 0.460).

**TABLE 2:** Corrected Akaike information criterion (AIC_c_) model selection results for the final model set from a successive model‐building procedure from daily nest survival models.

Model	*K*	AIC_c_	ΔAIC_c_
Shrub 300 + shrub 300^2^ + tree 30 + slope	6	567.27	0
Shrub 300 + shrub 300^2^ + tree 30 + slope + elevation + heat	8	567.64	0.37
Shrub 300 + shrub 300^2^ + tree 30	5	568.91	1.64
Shrub 300 + shrub 300^2^ + tree 40	5	569.34	2.07
Shrub 300 + shrub 300^2^ + tree 50	5	569.75	2.48
Shrub 300 + shrub 300^2^ + tree 60	5	570.23	2.96
Shrub 300 + shrub 300^2^ + tree 70	5	570.63	3.36
Shrub 300 + shrub 300^2^ + tree 80	5	570.89	3.62
Shrub 300 + shrub 300^2^ + tree 90	5	571.00	3.72
Shrub 300 + shrub 300^2^ + tree 100	5	571.17	3.90
Shrub 300 + shrub 300^2^	4	573.99	6.72
Shrub 300	3	575.34	8.08
Shrub 400 + shrub 400^2^	4	575.80	8.53
Shrub 400	3	576.91	9.64

*Note*: All models include year as a random effect.

Simulated fledgling production dropped from an average of 76.83 ± 4.89 (SD) fledglings km^−1^ historically to 49.72 ± 3.90 in the current time period (Figure 4C,D). In other words, we predict that fledgling production at the site dropped by roughly 35% from 662 in 1954 to 429 in 2020 due to increasing tree cover. The number of fledglings produced per territory was projected to decline to 23.04 ± 2.72 in the future *no action* scenario, corresponding to a total production of only 199 fledglings across the sites. In the future *restoration* scenario, we simulated that fledgling production was 53.34 ± 4.03 per territory, or a total of 460 fledglings.

## DISCUSSION

Our study quantifies the stark demographic consequences of conifer encroachment for a sagebrush‐obligate songbird, revealing a critical choice between proactive management and population decline. Over the past 70 years, our simulations suggest that the observed threefold increase in tree cover drove an estimated 30% decline in the Brewer's Sparrow population at our site. While the phenomenon of tree encroachment has long been documented (Foster, 1917; Miller, 1921), its role as a primary driver of wildlife population declines has only recently been recognized (Donovan et al., 2024). In fact, the threat of expanding tree cover went unmentioned in an influential review of conservation issues facing sagebrush avifauna 20 years ago (Knick et al., 2003). Our simulations show that continuing encroachment under a no scenario is predicted to cause a further 60% population loss over the next 30 years. Conversely, a strategy of sustained, targeted tree removal is predicted to nearly halt this decline, stabilizing both population size and fledgling production. Our findings thus provide a quantitative forecast of the potential population‐level outcomes of management action versus inaction, demonstrating that conifer encroachment is a primary and manageable threat to the persistence of sagebrush wildlife.

Given that trees have already encroached and infilled vast tracts of formerly open sagebrush habitat, our results demonstrate that conifer encroachment may play a larger role in the historical and ongoing losses of Brewer's Sparrow populations than previously recognized (Knick et al., 2003, 2011; Sauer et al., 2019). Invasive annual grasses and direct human modification of the landscape have long been thought of as the major drivers of wildlife declines in the sagebrush biome (Knick et al., 2003; Knick & Rotenberry, 1995; Sands et al., 1999). While these factors undoubtedly reduce sagebrush integrity, the precise impact of invasive annual grasses on rangeland bird populations has remained unclear despite multiple studies (Earnst & Holmes, 2012; Wiens & Rotenberry, 1985; Zeller et al., 2021). Additionally, direct human modification has caused only 3% of the overall loss of core sagebrush habitat since 2000, compared with 18% for conifer encroachment (Mozelewski et al., 2024). Given the complexities of converting cropland back into sagebrush habitat or battling invasive grasses, tree removal has emerged as a relatively simple restoration strategy.

Conifer removal, while simpler than other restoration methods, still requires forethought for successful implementation. Our study simulated the hand‐felling of encroaching trees, which preserves the shrub layer for wildlife, but is often impractical over large areas. Alternative management strategies, such as prescribed fires or mechanical removal, are more cost‐effective for large areas but have consequences (Ding et al., 2020; Ding & Eldridge, 2023b). Most methods of tree removal damage the sagebrush, causing abandonment by obligate wildlife until shrub regrowth. Additionally, large‐scale removal efforts can impact other ecosystem components, such as nutrient cycling, carbon storage, and forage production. Removal efforts must also ensure that at‐risk forest‐associated species, such as the Pinyon Jay, are not harmed (Reinhardt et al., 2023; Tack et al., 2023; Van Lanen et al., 2023). Finally, all methods of tree removal require continuous re‐treatment of new trees until more frequent fire cycles are restored to the landscape.

Our projections indicate that a *no action* scenario for conifer encroachment would result in a critical decline in the local Brewer's Sparrow population, but proactive restoration would nearly halt this decline. Often, encroaching trees are only seen as a problem once significant areas have transitioned to dense conifer forest. At this stage, the restoration of heavily forested sites is very costly and a successful return to sagebrush habitat is uncertain (Mozelewski et al., 2024). In our future scenario without restoration, 30 years of encroachment led to a sharp decline in shrub cover as trees shaded out sagebrush (Appendix S1: Figure S1; Coultrap et al., 2008; Roundy, Miller, et al., 2014). Dense tree cover displaces sagebrush songbirds in favor of edge‐ or forest‐associated species (Donovan et al., 2024; Reinkensmeyer et al., 2007; Zarri et al., 2024). While successional habitat change is a natural phenomenon, the larger context reveals much steeper range‐wide declines of sagebrush and grassland birds relative to woodland species (North American Bird Conservation Initiative, 2025; Rosenberg et al., 2019). Additionally, other sagebrush‐obligate wildlife have demonstrated even stronger avoidance of tree cover than Brewer's Sparrows, including Greater Sage‐Grouse, Sage Thrashers (*Oreoscoptes montanus*), and Sagebrush Sparrows (*Artemisiospiza nevadensis*; Baruch‐Mordo et al., 2013; Kumar et al., 2024; Zarri et al., 2024), indicating that tree removal can benefit many declining species.

While we found striking effects of tree cover on Brewer's Sparrow reproductive success, questions remain about the impact of tree encroachment on other vital rates. For example, trees have also encroached into the winter ranges of many sagebrush songbirds, but we have yet to fully understand the impact on overwinter survival. The vastness of the sagebrush biome, occupied by relatively few long‐term field stations, yields minimal data from traditional mark–recapture or mark–resight surveys (Sauer et al., 2019). Thus, we are still struggling to quantify the basic benchmarks of annual survival and site fidelity for many sagebrush songbirds, let alone the effect of tree removal treatment on these rates. As technological advances, such as small GPS tags and MOTUS tracking, become more accessible, future studies will be able to fill these knowledge gaps.

As efforts to remove encroaching trees increase, intact sagebrush ecosystems at greatest risk of conifer encroachment have already been identified for targeted restoration efforts (Reinhardt et al., 2023). Tree removal at a scale commensurate with the problem will require significant financial input and repeated treatment as trees continue to encroach. By demonstrating that targeted tree removal can halt population decline, our results, in conjunction with the priority maps from Reinhardt et al. (2023), provide a clear roadmap for efficient and effective conservation going forward.

## AUTHOR CONTRIBUTIONS

Elise C. Zarri, Jason D. Tack, Joseph T. Smith, Scott L. Morford, Thomas E. Martin, and David E. Naugle conceived the ideas and designed the methodology. Elise C. Zarri collected the data and led manuscript writing; and Elise C. Zarri, Jason D. Tack, Joseph T. Smith, and Scott L. Morford analyzed the data. All authors contributed critically to the drafts and gave final approval.

## CONFLICT OF INTEREST STATEMENT

The authors declare no conflicts of interest.

## Supporting information


Appendix S1.

## Data Availability

Data (Zarri et al., 2026) are available in Dryad at https://doi.org/10.5061/dryad.9s4mw6mx2.
