# Supplementary material for: Songbird population trajectories diverge under simulations of conifer encroachment versus removal in a sagebrush ecosystem
Source: Ecol Appl. 2026 Apr 6;36(3):e70228. doi: 10.1002/eap.70228 (PMC13051279; doi:10.1002/eap.70228)
Supplement: Supplementary file 1 — Appendix S1. [file EAP-36-e70228-s001.pdf]

Appendix S1

Songbird population trajectories diverge under simulations of conifer encroachment versus removal in a sagebrush ecosystem

Elise C. Zarri, Jason D. Tack, Joseph T. Smith, Scott L. Morford, Thomas E. Martin, David E. Naugle

Ecological Applications

Table S1. Model selection of Brewer's Sparrow clutch sizes relative to tree and shrub cover. Numbers after the model terms indicate the buffer size around the nest location. All models use Poisson error distributions.

Model	Tree		Shrub		K	AICc	ΔAICc
	Est. ± SE	p	Est. ± SE	p			
Tree 30 + Shrub 30	-0.0052 ± 0.012	0.66	0.0008 ± 0.006	0.99	3	936.04	0
Tree 300 + Shrub 300	0.0001 ± 0.006	0.98	0.0047 ± 0.011	0.68	3	936.07	0.03
Tree 40 + Shrub 40	-0.0045 ± 0.011	0.68	0.0002 ± 0.007	0.98	3	936.07	0.03
Tree 50 + Shrub 50	-0.0041 ± 0.010	0.69	0.0004 ± 0.007	0.95	3	936.08	0.04
Tree 1000 + Shrub 1000	0.0023 ± 0.006	0.70	0.0100 ± 0.026	0.70	3	936.08	0.04
Tree 200 + Shrub 200	-0.0008 ± 0.007	0.91	0.0034 ± 0.009	0.72	3	936.08	0.04
Tree 60 + Shrub 60	-0.0038 ± 0.010	0.70	0.0006 ± 0.007	0.94	3	936.08	0.04
Tree 70 + Shrub 70	-0.0034 ± 0.009	0.72	0.0007 ± 0.007	0.92	3	936.09	0.05
Tree 80 + Shrub 80	-0.0030 ± 0.009	0.74	0.0009 ± 0.007	0.91	3	936.10	0.07
Tree 90 + Shrub 90	-0.0026 ± 0.009	0.76	0.001 ± 0.008	0.90	3	936.12	0.08
Tree 100 + Shrub 100	-0.0022 ± 0.008	0.79	0.001 ± 0.008	0.88	3	936.14	0.10
Tree 400 + Shrub 400	0.0006 ± 0.005	0.91	0.004 ± 0.014	0.77	3	936.16	0.12
Tree 500 + Shrub 500	0.0008 ± 0.005	0.88	0.003 ± 0.017	0.87	3	936.21	0.17

Table S2. Model selection of the proportion of Brewer's Sparrow eggs that successfully fledge. All models use binomial error distributions.

Model	Tree		Shrub		K	AICc	ΔAICc
	Est. ± SE	p	Est. ± SE	p			
Tree 30 + Shrub 30	0.029 ± 0.090	0.74	-0.034 ± 0.045	0.44	3	143.73	0
Tree 40 + Shrub 40	0.026 ± 0.085	0.76	-0.035 ± 0.046	0.45	3	143.87	0.13
Tree 300 + Shrub 300	-0.015 ± 0.036	0.67	-0.070 ± 0.082	0.39	3	144.00	0.27
Tree 200 + Shrub 200	-0.003 ± 0.043	0.95	-0.056 ± 0.067	0.40	3	144.03	0.29
Tree 50 + Shrub 50	0.021 ± 0.079	0.79	-0.035 ± 0.047	0.46	3	144.05	0.32
Tree 400 + Shrub 400	-0.025 ± 0.033	0.45	-0.088 ± 0.103	0.39	3	144.33	0.60
Tree 60 + Shrub 60	0.012 ± 0.072	0.87	-0.035 ± 0.048	0.46	3	144.36	0.62
Tree 70 + Shrub 70	0.006 ± 0.066	0.93	-0.036 ± 0.049	0.47	3	144.55	0.82
Tree 1000 + Shrub 1000	-0.036 ± 0.042	0.39	-0.135 ± 0.199	0.50	3	144.68	0.95
Tree 80 + Shrub 80	0.002 ± 0.063	0.97	-0.035 ± 0.050	0.48	3	144.75	1.02
Tree 90 + Shrub 90	-0.001 ± 0.060	0.99	-0.034 ± 0.051	0.50	3	144.94	1.21
Tree 500 + Shrub 500	-0.027 ± 0.033	0.41	-0.077 ± 0.128	0.55	3	145.00	1.27
Tree 100 + Shrub 100	-0.003 ± 0.057	0.96	-0.034 ± 0.053	0.51	3	145.03	1.29

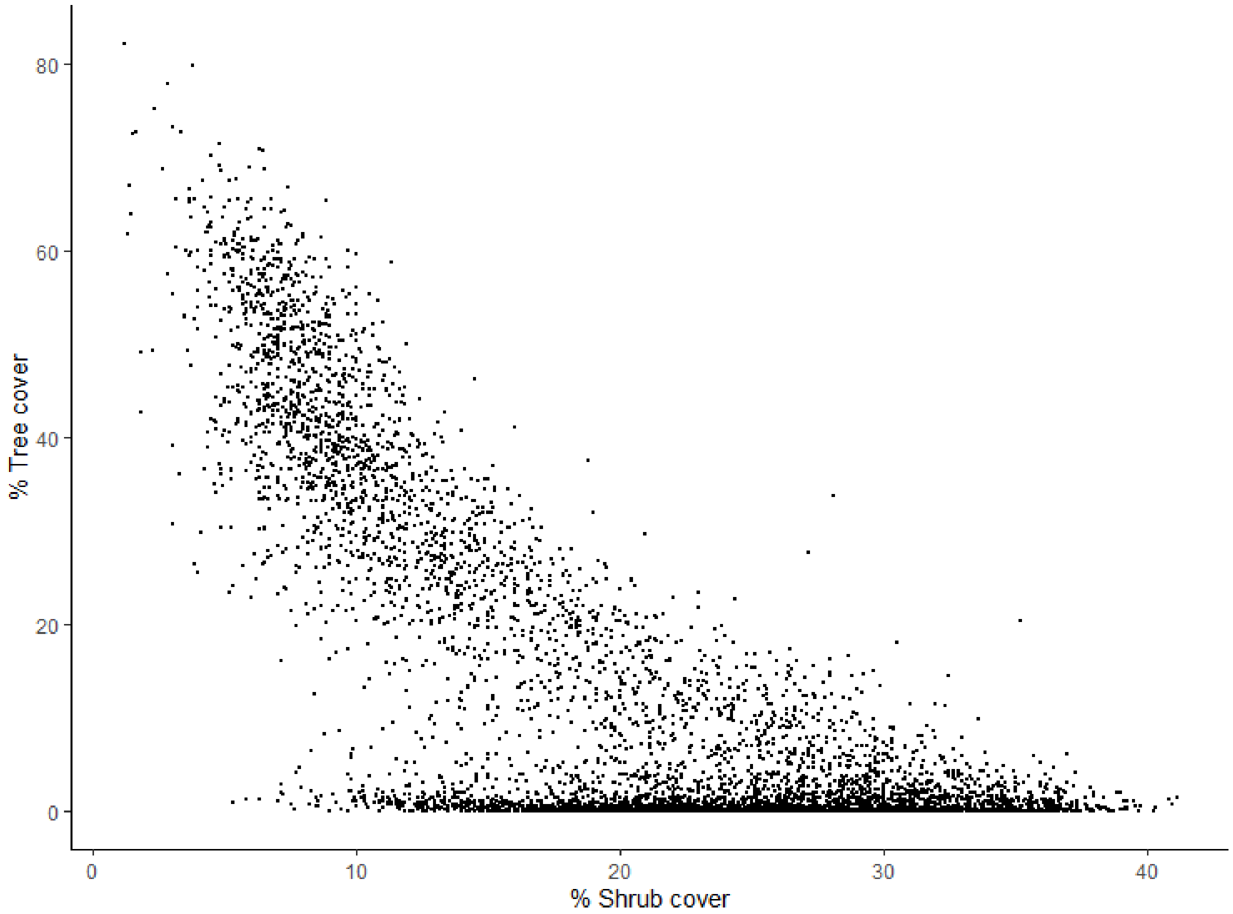

Figure S1. Grid cell data depicting tree and shrub cover at the study site in 2019–2022. Data extracted at 70 × 70 m resolution from the Rangeland Analysis Platform.
